# Continuous Glucose Monitoring in Newly Diagnosed Type 2 Diabetes Patients Reveals a Potential Risk of Hypoglycemia in Older Men

**DOI:** 10.1155/2017/2740372

**Published:** 2017-02-08

**Authors:** Feng-fei Li, Bing-li Liu, Hong-hong Zhu, Ting Li, Wen-li Zhang, Xiao-fei Su, Jin-dan Wu, Xue-qin Wang, Ning Xu, Wei-Nan Yu, Qun Yuan, Guan-cheng Qi, Lei Ye, Kok-Onn Lee, Jian-hua Ma

**Affiliations:** ^1^Department of Endocrinology, Nanjing First Hospital, Nanjing Medical University, Nanjing, China; ^2^Department of Endocrinology, The First People's Hospital of Nantong, Nantong, China; ^3^Department of Endocrinology, The First People's Hospital of Lianyungang, Lianyungang, China; ^4^Department of Endocrinology and Metabolism, Huai'an Hospital Affiliated to Xuzhou Medical College and Huai'an Second People's Hospital, Huai'an, China; ^5^Department of Endocrinology, Changzhou Hospital of Traditional Chinese Medicine, Changzhou, China; ^6^Department of Endocrinology, Lianyungang Oriental Hospital, Lianyungang, China; ^7^National Heart Research Institute Singapore, National Heart Centre Singapore, Singapore; ^8^Department of Medicine, National University of Singapore, Singapore

## Abstract

*Objectives. *We performed continuous glucose monitoring (CGM) to define the features of patients with newly diagnosed type 2 diabetes (T2D) before and after Continuous Subcutaneous Insulin Infusion (CSII) therapy.* Methods. *This was a retrospective analysis. Newly diagnosed T2D patients (106) were admitted from eight centers in China. They were divided into a younger patient group (<60 years) and an older patient group (≥60 years). Each group was further divided into male and female patients. CSII therapy was maintained for 3 weeks after the glycemic target was reached. CGM was performed 2 times before and after completion of insulin treatment.* Results. *CGM data showed the expected significant improvement of mean amplitude glycemic excursion (MAGE) with CSII therapy. The older patients had lower hourly glucose concentrations from 0200 to 0700 o'clock compared to the younger patients at baseline. Surprisingly, in the older patient group, the male patients had a potential risk of hypoglycemia after CSII therapy, especially during periods from 2300 to 2400 and 0400 to 0600.* Conclusions. *Our data suggested that older male patients with newly diagnosed T2D may have lower nocturnal glucose concentrations. This may potentially increase the risk of nocturnal hypoglycemia during CSII therapy. This study was registered with Chinese Clinical Trial Registry, number CliCTR-TRC-11001218.

## 1. Introduction

Intensive insulin therapy has been used to treat patients with type 2 diabetes (T2D) in China [1]. A proportion of patients with longstanding type 2 diabetes mellitus respond well to intensive treatment [2] and are able to maintain euglycemia with minimal treatment for a long period [3]. Indeed, patients' response to insulin intensive therapy is quietly variable, mirroring the heterogeneity of diabetes [2]. A relatively large study did highlight the increased risk of hypoglycemia in the older patients but did not differentiate between the male and female patients [4, 5]. Reports have highlighted the importance of hypoglycemia, defined as glucose concentrations less than 4.0 mmol/L, in intensive insulin therapy compared to conventional therapy with or without the use of an insulin pump [6]. The Diabetes Control and Complications Trial (DCCT) study demonstrated that nearly 20% of patients receiving insulin pump therapy had symptomless nocturnal hypoglycemia [7]. Hypoglycemia, especially nocturnal hypoglycemia, might be an important barrier for patients with diabetes to achieve better glucose control or even resulting in death in severe cases [8]. It is therefore important to identify potential factors and associations for hypoglycemia during intensive insulin therapy [9].

However, there have been no previous studies in Chinese patients where continuous glucose monitoring (CGM) has been used to define the features of such patients before and after treatment. In the current study, we performed 2 times 3-day CGM on all consecutive patients with newly diagnosed T2D before and after intensive insulin therapy to characterize the features of 24-hour plasma glucose profile.

## 2. Methods

Between February 2010 and December 2014, we recruited a total of 106 patients with newly diagnosed T2D from 8 hospitals in Jiangsu province in China. The inclusion criteria were (1) patients aged between 18 and 80 years and (2) 9.0% < HbA1c ≤ 12% at diagnosis. Patients were excluded from analysis if they had chronic kidney disease, if they were positive for antiglutamic acid decarboxylase (aGAD) antibody, or if they had maturity onset diabetes in the young (MODY) or mitochondria diabetes mellitus [1]. The study protocol and patient consent forms were approved by the Institutional Ethical Committee of Nanjing First Hospital, Nanjing Medical University, the First People's Hospital of Nantong, the First People's Hospital of Lianyungang, Huai'an Second People's Hospital, Changzhou Hospital of Traditional Chinese Medicine, and Lianyungang Oriental Hospital. All patients gave written informed consent. The methods were carried out in accordance with the Declaration of Helsinki guidelines, including any relevant details.

We recruited all drug naïve patients with newly diagnosed T2D and HbA1c concentrations > 9%. They did not receive any medical treatment before the assessment of initial CGM in the study. All these patients were recruited to receive CSII after informed consent was obtained. All patients were admitted as inpatients for Continuous Subcutaneous Insulin Infusion (CSII) therapy and CGM. After the baseline parameters were assessed, fasting blood serum was collected for insulin and C-peptide measurements. Enrolled subjects received intensive insulin treatment, without any oral antidiabetic drugs except metformin. Patients were divided into two groups based on their age: younger patient group: <60 years and older patient group: ≥60 years (the older population in China was above 60 years old). Patients in each group were also divided into two subgroups based on their gender (Table 1). The total daily insulin (human insulin, Novo Nordisk, Bagsværd, Denmark) dose was 0.5 IU/kg which was given in two modes: 50% of total daily dose was given as boluses with three meals at a fixed rate, and the remaining insulin was given over 24 hours (hrs). Insulin doses were then titrated on an individual-patient basis using the algorithm (if the fasting blood glucose level was less than 4.4 mmol/L, the basal insulin dose was reduced 0.2 units per hour from 1900 to 0500 by slowing the infusion speed; if the fasting blood glucose level was within 4.4 to 6.1 mmol/L, the basal insulin dose would be unchanged; if the fasting blood glucose level was from 6.2 to 7.8 and 7.9 to 10.0 and >10.0 mmol/L, the basal insulin dose would be increased subsequently by 0.2, 0.4, and 0.6 units per hour from 1900 to 0500 by increasing the infusion speed, resp., and if 2-hour postprandial blood glucose level was up, then the bolus insulin dose was titrated with the same algorithm as basal dose). When euglycemic control was achieved, treatments would be unchanged for another 3 weeks.

All patients were subjected to 2 times' 3-day retrospective CGM (Sof-sensor, CGMS-Gold, Medtronic Incorporated, Northridge, USA) in hospital by the specialist nurse during the study period. The first CGM was performed before therapy, and the second CGM was done after the completion of insulin treatment. Briefly, the CGM sensor was subcutaneously embedded at Day 0 around 1600–1700 PM. If CGM was performing well, subjects were instructed to keep the sensor fixed. The study nurse inputted at least 4 calibration readings every day. At Day 4, around 1600–1700 PM, subjects had the sensor removed, and the CGM data were saved by the investigator, as described previously [10–12]. All patients received the same energy intake during the CGM periods. All subjects were instructed to maintain their usual physical activity and received meals consisting of a total daily caloric intake of 25 kcal/kg/day. The ratio of carbohydrate, proteins, and fats was 55%, 17% and 28%, respectively.

The 24-hrs mean glucose (MG), the standard deviation of the MG, the coefficient of variation (CV), the 24-hrs mean amplitude of glycemic excursions (MAGE), the percentage time duration (%) of hyperglycemia (glucose > 10.0 mmol/L), the incremental area under curve (AUC) of glucose above 10.0 mmol/L, and the hourly BG were recorded and calculated. The *β*-cell function and insulin resistance were assessed by the homoeostasis model assessment B (HOMA-B) and HOMA-IR [1, 13]. Matsuda Index [14] and Insulinogenic Index [15] were calculated as previously described.

Insulin, C-peptide, and HbA1c were measured centrally at the Department of Endocrinology, Nanjing First Hospital, Nanjing Medical University. Routine clinical laboratory tests were done in the central laboratory of the eight participating centers.

### 2.1. Statistical Analysis

Data were analyzed with the SPSS PASW Statistics 18 Package. Shapiro-Wilk test was used to assess the distribution of data. Normally distributed and continuous variables are presented as mean ± standard deviation (SD). Non-normally distributed variables were presented as median (IQR) and logarithmically transformed before analysis. The independent samples *t*-test was used to compare the differences between two groups. A two-way ANOVA for repeated measurements was used in the comparison between groups. Bonferroni correction was followed. *P* values were two-tailed with a significance level of 5%.

## 3. Results

Consecution of 106 newly diagnosed T2D patients met inclusion criteria (58 men and 48 women, age 53.50 ± 9.84 years, body-mass index 24.85 ± 3.20 kg/m^2^, HbA_1c_  9.92 ± 1.83%, and mean fasting plasma glucose 10.84 ± 2.88 mmol/L) and were admitted to the study. We excluded a total of 2 younger patients (1 male and 1 female) who had glucose levels more than 22.2 mmol/L during the first CGM period as the CGM sensor used in this study is unable to monitor glucose concentrations > 22.2 mmol/L.

Patients were then divided into two groups. There were no differences in demographic characteristics between the 69 younger patients and the 35 older patients (Table 1). The younger female patients had higher 2-hr postprandial insulin concentrations, but all other baseline characteristics were similar between male and female patients in the younger patient group. The baseline characteristics were similar between male and female older patients.

**Figure 1:**
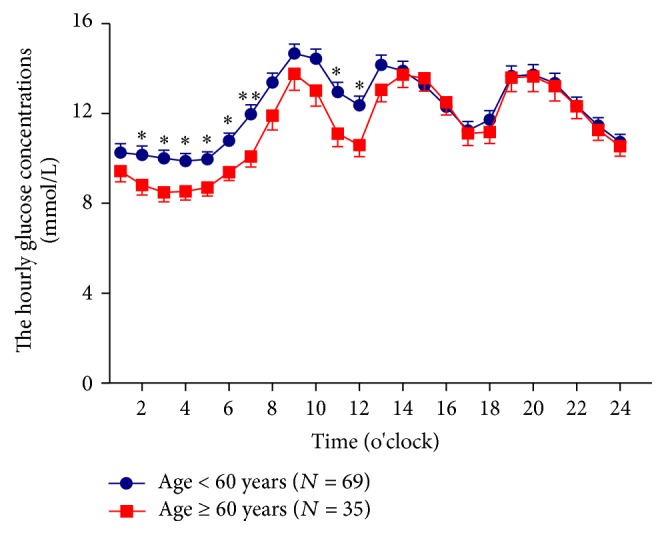
The hourly glucose concentrations in older patients (≥60 years) and younger patients (<60 years). Data are presented as means ± SD. A two-way ANOVA was used in the comparison between groups, ^*∗*^*P* < 0.05 and ^*∗∗*^*P* < 0.01.

There were no differences in the 24-hrs MG, the SD of 24-hrs MG, the CV of 4-hrs MG, the MAGE, the time spent in hyperglycemia (>10 mmol/L) and the incremental AUC of hyperglycemia, and the percentage time duration (%) of hypoglycemia (glucose > 10.0 mmol/L) within the two groups. CGM data showed that the hourly glucose concentrations (mmol/L) in older patients were lower than that in younger patients, especially from 0200 to 0700 o'clock (8.94 ± 2.69, 8.60 ± 2.50, 8.63 ± 2.26, 8.77 ± 2.18, 9.46 ± 2.18, and 10.13 ± 2.75 versus 10.16 ± 3.53, 10.01 ± 3.26, 9.88 ± 3.04, 9.97 ± 2.79, 10.79 ± 3.03, and 11.97 ± 3.68, *P* < 0.05, resp.) and 1100 to 1200 o'clock (11.11 ± 3.47, 10.60 ± 3.11 versus 12.96 ± 3.82, 12.37 ± 3.45, *P* < 0.05, resp.) (Figure 1).

The same standard weight based intensive insulin therapy treatment protocol was applied to all patients. CGM data showed that, as expected, all patients had significant improvement of MAGE, 24-hrs MG, and the incremental AUC of glucose above 10.0 mmol/L after treatment with intensive insulin therapy. No statistically significant differences in the abovementioned parameters were found when comparing between the groups before treatment. However, the female patients exhibited an increased 24-hrs MG levels after CSII treatment compared to the male patients using the same insulin therapy treatment protocol (Table 2). The younger male and female 24-hrs MG levels were almost the same after CSII treatment (7.3 ± 1.1 versus 7.7 ± 1.3, *P* = 0.13).

**Figure 2:**
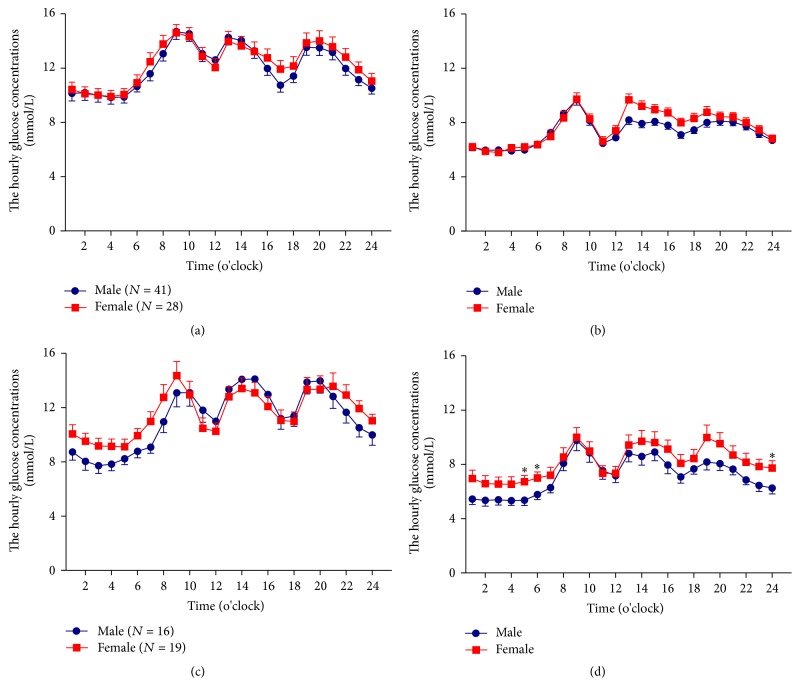
The hourly glucose concentrations calculated from CGM. The hourly glucose concentrations in young patients (<60 years) before (a) and after intensive insulin therapy treatment (b). The hourly glucose concentrations in older patients (≥60 years) before (c) and after intensive insulin therapy treatment (d). Data are presented as means ± SD. A two-way ANOVA was used in the comparison between groups, ^*∗*^*P* < 0.05.

A stratified analysis comparing the older male and female groups revealed that male patients experienced lower glucose concentrations during intensive insulin therapy compared to female patients. In contrast, the younger male and female patient subgroups (<60 years) did not differ and had similar mean glucose concentrations at every hour at baseline before and after intensive insulin therapy (Figures 2(a) and 2(b)).

There was a tendency of lower glucose concentrations in older male subgroup as compared with that of old female subgroup at baseline, especially from 2200 to 0500 (Figure 2(c)), but this tendency was not statistically significant. After 3-week intensive insulin therapy, the hourly glucose concentrations, especially between 2300 and 2400 and from 0400 to 0600, in older male patient subgroup were significantly lower compared with that of the older female patients (*P* < 0.05, resp.) (Figure 2(d)). Furthermore, the 0000–0600 hours' glucose concentrations in older male patients were significantly lower than that of older female patients after CSII treatment (5.56 ± 1.38 versus 6.96 ± 2.14 mmol/L, *P* = 0.032). In addition, we did not observe statistic differences in nocturnal glycemic variations (0000–0600 hours) between old male and female patient groups after treatment (SD: 0.79 ± 0.31 versus 0.94 ± 0.56 mmol/L, *P* = 0.341; and CV: 0.15 ± 0.07 versus 0.13 ± 0.07, *P* = 0.452).

Our data thus demonstrated that the older (≥60 years of age) male patients with newly diagnosed T2D had significantly lower mean plasma glucose concentration as compared with female patients during the nocturnal period. The lower mean plasma glucose concentration was more prominent at some isolated time points during intensive insulin treatment.

## 4. Discussion

Our data revealed a novel observation that older men with newly diagnosed T2D in Jiangsu Provence in China have a high incidence of nocturnal low glucose before and during intensive insulin therapy. Our data indicates that special attention should be paid to prevention of hypoglycemia in elderly male patients when started on intensive insulin therapy with insulin pump.

The number of older patients with diabetes started on intensive insulin treatment has been increasing in recent years [16]. This has mainly been in type 1 diabetes mellitus patients. Few studies have used intensive insulin therapy in older patients with T2D. A relatively large study did highlight the increased risk of hypoglycemia in the older age group but did not differentiate between the male and female patients [4, 5]. In the pilot study, the same standard weight based intensive insulin therapy treatment protocol was applied to all patients. Our data strengthens this finding and further indicates that older patients with newly diagnosed T2D, especially older men in the current study population, were prone to hypoglycemia especially in nocturnal time.

The ACCORD study, which aimed to determine whether intensive therapy to treat to the target of normal glycated hemoglobin levels would reduce cardiovascular events in patients with T2D, found that intensive-therapy group had a significantly higher mortality as compared with standard therapy group [17]. The mean age of study subjects was 53.5 years and 46% of them are male patients. Hypoglycemia requiring medical assistance was much more frequent in the intensive-therapy group. Our current study is partially consistent with their findings [17], although the low glucose did not reach the criteria of significant hypoglycemia during their short hospital study period. Our data suggests that this may be important in intensive insulin therapy with or without using an insulin pump.

China has tremendous T2D patients according to national survey performed by Yang et al. in 2010 [18]. Increasing evidences demonstrated that patients with T2D in Chinese population are quite different from the Western countries, such as the thrifty gene carried in Chinese population [19], the different pattern of intake of nutrients and life style, the good responsiveness to oral antidiabetic agents (e.g., *α*-glucosidase inhibitor and sulfonylureas), the lower insulin dose requirements, the higher remission rate of short intensive insulin therapy [20], and the lower BMI and the less waist circumferences compared to whites [21]. In addition, Chinese population has the higher percentage of body fat than Europeans and African Americans at the same level of BMI [22, 23]. Early intensive insulin therapy in patients with newly diagnosed T2D in Chinese population achieved prolonged glycemic remission, as well as recovery and maintenance of *β*-cell function compared with treatment with oral hypoglycemic agents [1]. However, the importance of hypoglycemia in intensive insulin therapy compared to conventional therapy should be paid more attention.

Our data also indicated that patients with onset of T2D had relatively less BMI (24.85 ± 3.20 kg/m^2^). This was in agreement with Ntuk et al. who reported that Chinese women with BMI 24.0 kg/m^2^ and Chinese men with BMI 26.0 kg/m^2^ have the equivalent prevalence of diabetes at BMI 30 kg/m^2^ in Western participants [21]. Furthermore, we found that insulin resistance was elevated in patients with higher BMI (≥25 kg/m^2^) compared to patients with less BMI (<25 kg/m^2^) [3.24 (2.25, 5.75) versus 2.05 (1.27, 3.37), *P* < 0.01]. We did not exclude patients with BMI less than 20 kg/m^2^, for Stommel and Schoenborn revealed that the chronic disease risk in underweight people was not increased compared with persons with BMI in the range of 20-21 and analyzed a very large sample (337,375 adults) [24].

Our study has several limitations. First, the study only observed patients population in Jiangsu Provence in China, so the situation might not be applicable to patients of other populations. Second, the stratified sample size (the older male and female patients) was relatively modest. Third, we did not demonstrate the underline mechanisms of older male patients who had a higher risk of nocturnal hypoglycemia when started on insulin intensive therapy. However, the increase of Matsuda Index (insulin sensitivity) [25] in male older patients compared to female older patients in our study might be related to the potential of nocturnal hypoglycemia in older male patients.

It is unknown why the old Chinese patients (≥60 years) tend to have higher incidence of lower nocturnal glucose concentrations, which may be potential risk of nocturnal hypoglycemia, especially after intensive insulin therapy. However, this highlights that more careful blood glucose monitoring may be necessary in older patients receiving intensive insulin therapy, especially in men, to prevent the incidence of nocturnal hypoglycemia.

## Figures and Tables

**Table 1:** Demographic characteristics in study subjects. Data are presented as means ± SD. *t*-test was used to compare the differences between two groups, ^*∗*^*P* < 0.05.

Items	Total patients			Total patients			Younger (age < 60 years)			Older (age ≥ 60 years)		
	Male (57)	Female (47)	*P* value	Younger (69)	Older (35)	*P* value	Male (41)	Female (28)	*P* value	Male (16)	Female (19)	*P* value
Age (years)	51.7 ± 10.5	55.7 ± 8.9	0.06	48.7 ± 7.5	64.5 ± 4.1	0.00	47.3 ± 8.2	50.6 ± 6.2	0.06	64.5 ± 3.4	64.6 ± 4.7	0.96
BMI (kg/m^2^)	24.7 ± 3.1	24.9 ± 3.4	0.71	25.0 ± 3.1	24.4 ± 3.5	0.43	24.6 ± 2.7	25.4 ± 3.6	0.28	24.9 ± 3.9	24.0 ± 3.0	0.50
HbA1c (%)	10.0 ± 1.8	9.8 ± 1.8	0.65	10.1 ± 1.8	9.3 ± 1.7	0.30	10.1 ± 1.8	10.2 ± 1.8	0.89	9.5 ± 1.9	9.1 ± 1.6	0.51
FPG	10.6 ± 2.9	10.8 ± 2.6	0.72	11.0 ± 2.9	10.0 ± 2.3	0.08	11.0 ± 3.0	11.1 ± 2.7	0.83	9.7 ± 2.1	10.3 ± 2.5	0.41
LnFPI^†^	1.6 ± 0.6	1.8 ± 0.8	0.11	1.8 ± 0.7	1.6 ± 0.8	0.15	1.7 ± 0.6	1.9 ± 0.8	0.12	1.4 ± 0.8	1.7 ± 0.7	0.40
Ln2-PPI^†^	2.8 ± 0.8	3.2 ± 0.9	0.02^*∗*^	3.0 ± 0.8	2.8 ± 0.9	0.07	3.0 ± 0.8	3.3 ± 0.9	0.10	2.4 ± 0.7	3.1 ± 0.9	0.04
FC	2.1 ± 0.9	2.4 ± 1.3	0.22	2.4 ± 1.2	1.9 ± 1.0	0.06	2.2 ± 0.9	2.6 ± 1.5	0.23	1.8 ± 1.0	2.1 ± 0.9	0.44
2-h FC	4.9 ± 2.3	6.0 ± 2.8	0.03^*∗*^	5.5 ± 2.6	5.2 ± 2.6	0.54	5.1 ± 2.3	6.1 ± 2.9	0.10	4.5 ± 2.5	5.8 ± 2.6	0.15
LnHOMA-IR^†^	2.9 ± 2.5	4.0 ± 3.6	0.07	3.6 ± 2.9	3.0 ± 3.5	0.38	2.9 ± 1.5	4.4 ± 4.0	0.02^*∗*^	2.9 ± 4.3	3.1 ± 2.7	0.84
LnHOMA-B^†^	21.7 ± 22.8	26.0 ± 24.2	0.33	24.7 ± 25.2	21.7 ± 19.2	0.55	22.1 ± 23.0	28.0 ± 28.0	0.32	20.8 ± 23.1	22.6 ± 15.5	0.79
II	1.2 ± 1.3	1.6 ± 1.8	0.17	1.2 ± 1.6	1.8 ± 1.6	0.10	1.1 ± 1.2	1.5 ± 2.0	0.19	1.8 ± 1.7	1.8 ± 1.5	0.91
MI	130.8 ± 93.8	100.4 ± 90.3	0.09	127.8 ± 100.0	93.4 ± 53.4	0.08	140.0 ± 100.7	108.6 ± 107.8	0.06	115.3 ± 58.13	75.9 ± 24.3	0.02^*∗*^
TI	36.3 ± 16.6	40.2 ± 19.8	0.37	38.2 ± 19.8	38.3 ± 14.1	0.97	38.3 ± 18.4	43.5 ± 22.8	0.38	31.1 ± 9.8	33.1 ± 6.9	0.59
Basal	15.4 ± 7.4	16.8 ± 8.0	0.45	15.8 ± 8.1	16.7 ± 6.8	0.68	15.7 ± 7.6	16.0 ± 8.7	0.89	14.7 ± 7.2	18.5 ± 6.2	0.20
Bolus	25.1 ± 13.6	26.1 ± 17.9	0.80	26.2 ± 16.7	24.1 ± 13.6	0.61	25.9 ± 15.1	26.5 ± 18.6	0.91	22.9 ± 9.2	25.2 ± 17.0	0.71

^†^After log transformation for non-normally distributed data. Data are presented as means ± SD. ^*∗*^*P* < 0.05, female patients versus male patients. FPG: fasting plasma glucose (mmol/L), LnFPG: Ln fasting plasma insulin (mU/L), Ln2-PPI:Ln 2-h postprandial insulin (mU/L), FC: fasting plasma C-peptide (pmol/L), 2-hFC: 2-h postprandial C-peptide (pmol/L), MI: Matsuda Index, II: Insulinogenic Index, TI: the total insulin doses per day (IU), Basal: basal insulin dose (IU), and Bolus: bolus insulin dose (IU).

**Table 2:** Glycemic variations in study subjects before and after intensive insulin therapy treatment. Data are presented as means ± SD. *t*-test was used to compare the differences between two groups, ^*∗*^*P* < 0.05.

Items	Total patients			Total patients			Younger (age < 60 years)			Older (age ≥ 60 years)		
	Male (57)	Female (47)	*P* value	Younger (69)	Older (35)	*P* value	Male (41)	Female (28)	*P* value	Male (16)	Female (19)	*P* value
MAGE before CSII	6.7 ± 2.7	6.4 ± 3.1	0.55	6.7 ± 3.1	6.3 ± 2.4	0.47	6.7 ± 2.8	6.7 ± 3.5	0.99	6.8 ± 2.3	5.9 ± 2.4	0.27
MAGE after CSII	3.0 ± 1.9	2.9 ± 2.0	0.9	3.1 ± 2.0	2.6 ± 1.8	0.21	3.1 ± 1.9	3.1 ± 2.1	0.94	2.6 ± 2.0	2.6 ± 1.7	0.94
MG before CSII	11.9 ± 2.6	12.1 ± 2.6	0.63	12.2 ± 2.7	11.4 ± 2.4	0.14	12.1 ± 2.6	12.3 ± 2.7	0.68	11.2 ± 2.3	11.6 ± 2.5	0.58
MG after CSII	7.3 ± 1.2	7.9 ± 1.7	0.02^*∗*^	7.5 ± 1.2	7.8 ± 2.0	0.26	7.3 ± 1.1	7.7 ± 1.3	0.13	7.2 ± 1.4	8.3 ± 2.3	0.11
SD before CSII	2.70 ± 0.96	2.58 ± 0.85	0.53	2.64 ± 0.90	2.61 ± 0.94	0.88	2.62 ± 0.96	2.67 ± 0.84	0.84	2.85 ± 0.98	2.41 ± 0.88	0.18
SD after CSII	1.77 ± 0.61	1.90 ± 0.75	0.31	1.81 ± 0.66	1.88 ± 0.73	0.59	1.72 ± 0.61	1.93 ± 0.71	0.16	1.92 ± 0.61	1.85 ± 0.84	0.78
CV before CSII	0.23 ± 0.08	0.22 ± 0.07	0.32	0.22 ± 0.08	0.23 ± 0.08	0.66	0.22 ± 0.08	0.22 ± 0.08	0.99	0.26 ± 0.08	0.21 ± 0.07	0.06
CV after CSII	0.24 ± 0.07	0.24 ± 0.08	0.86	0.24 ± 0.07	0.24 ± 0.07	0.87	0.23 ± 0.07	0.25 ± 0.08	0.36	0.27 ± 0.06	0.22 ± 0.08	0.10
AUC before CSII	2.65 ± 2.09	2.59 ± 1.93	0.88	2.75 ± 2.08	2.33 ± 1.85	0.3	2.8 ± 2.2	2.6 ± 2.	0.67	2.1 ± 1.8	2.5 ± 1.9	0.53
AUC after CSII	0.21 ± 0.32	0.32 ± 0.55	0.19	0.22 ± 0.32	0.35 ± 0.64	0.15	0.2 ± 0.3	0.3 ± 0.4	0.38	0.3 ± 0.4	0.4 ± 0.8	0.45
PT (%) before CSII	63.6 ± 28.1	64.4 ± 32.7	0.89	65.4 ± 30.2	60.6 ± 30.0	0.43	66.5 ± 29.0	64.0 ± 32.2	0.72	55.2 ± 23.9	65.2 ± 34.3	0.34
PT (%) after CSII	11.3 ± 12.8	16.0 ± 16.5	0.09	12.5 ± 13.3	15.6 ± 17.4	0.31	10.9 ± 11.8	14.9 ± 15.1	0.19	12.7 ± 15.6	18.0 ± 18.8	0.38

Data are presented as means ± SD. ^**∗**^*P* < 0.05, female patients versus male patients. MAGE: mean amplitude of glycemic excursions (mmol/L), MG: mean glucose (mmol/L), SD: standard deviation (mmol/L), CV: coefficient of variation (%), AUC: area under curve above 10.0 mmol/L (mmol/L per day), PT: the percentage time duration of glucose above 10.0 mmol/L (%).
